# Synergistically Acting on Myostatin and Agrin Pathways Increases Neuromuscular Junction Stability and Endurance in Old Mice

**DOI:** 10.14336/AD.2023.0713-1

**Published:** 2024-04-01

**Authors:** Roberta Schellino, Marina Boido, Jan W Vrijbloed, Ruggero G Fariello, Alessandro Vercelli

**Affiliations:** ^1^Department of Neuroscience Rita Levi-Montalcini, University of Turin, Turin 10126, Italy; ^2^Neuroscience Institute Cavalieri Ottolenghi, University of Turin, Orbassano, 10043 Italy; ^3^PharmaFox Therapeutics AG, Möhlin, Aargau, Switzerland

**Keywords:** aging, sarcopenia, neuromuscular junction, muscle innervation, endurance

## Abstract

Sarcopenia is the primary cause of impaired motor performance in the elderly. The current prevailing approach to counteract such condition is increasing the muscle mass through inhibition of the myostatin system: however, this strategy only moderately improves muscular strength, not being able to sustain the innervation of the hypertrophic muscle per se, leading to a progressive worsening of motor performances. Thus, we proposed the administration of ActR-Fc-nLG3, a protein that combines the soluble activin receptor, a strong myostatin inhibitor, with the C-terminal agrin nLG3 domain. This compound has the potential of reinforcing neuro-muscular stability to the hypertrophic muscle. We previously demonstrated an enhancement of motor endurance and ACh receptor aggregation in young mice after ActR-Fc-nLG3 administration. Now we extended these observations by demonstrating that also in aged (2 years-old) mice, long-term administration of ActR-Fc-nLG3 increases in a sustained way both motor endurance and muscle strength, compared with ActR-Fc, a myostatin inhibitor, alone. Histological data demonstrate that the administration of this biological improves neuromuscular stability and fiber innervation maintenance, preventing muscle fiber atrophy and inducing only moderate hypertrophy. Moreover, at the postsynaptic site we observe an increased folding in the soleplate, a likely anatomical substrate for improved neurotransmission efficiency in the NMJ, that may lead to enhanced motor endurance. We suggest that ActR-Fc-nLG3 may become a valid option for treating sarcopenia and possibly other disorders of striatal muscles.

## INTRODUCTION

Sarcopenia is the primary cause of motor disability in the elderly being also responsible for increased morbidity and mortality [1]. In the past decades, several attempts have been made to devise strategies to counteract the onset and progression of decaying muscle mass, strength and performance that characterize sarcopenia. Additionally, a great number of muscle pathologies that affect individuals from birth to senility exhibit a wide range of severity, from moderate impairment of muscle performance to rapidly progressing diseases that lead to complete immobility and death [2, 3].

A prevailing approach to ameliorate muscle function has been the manipulation of the myostatin system [4-8]. Myostatin is a member of the transforming growth factor β (TGFβ) superfamily. The Activin/Myostatin/TGFβ group binds plasma membrane-associated activin type IIB and type IIA receptors (ActRIIB/IIA) and, through the recruitment and activation of different activin receptor-like kinases, exerts a negative regulator function on muscle growth [9]. The myostatin inhibition approach has succeeded in increasing muscle mass and moderately also strength [10,11], but universally failed to ameliorate endurance both experimentally and in clinical trials [12 -15]. In fact, muscle trophism is a complex and multifarious process. Thus, we reasoned that addressing the performance deficit typical of sarcopenia and neuromuscular disorders by just targeting the muscle, as the myostatin block does, should not be sufficient, given the important role played by altered innervation in interfering with muscle function [16].

Our working hypothesis is that a dual action directed at both muscle and nerve, particularly targeting the neuromuscular junction (NMJ), a site known to deteriorate with aging [17-19], would be necessary for correcting the performance deficit seen in sarcopenia. For this reason, we focus on the role of agrin in the synaptic cleft, since the deletion of agrin from adult motor neurons resulted in both loss of acetylcholine (ACh) receptors and nerve terminal withdrawal from muscle fibers [20].

Thus, here we tested on 22 month-old mice a biological compound, ActR-Fc-nLG3, that combines the nLG3 domain from the C-terminus of human agrin (for its well-known role as a promoter of NMJs formation and maintenance [21, 22]) with the potent myostatin inhibitor ActR-Fc, via the constant region of an Igg1 monoclonal antibody. We previously demonstrated that the administration of ActR-Fc-nLG3 to young mice resulted in a moderate increase in muscle mass, an increase in strength comparable to the one seen with myostatin inhibitors, but also a notable enhancement of motor performance in the rotarod [23].

In the present work, we expanded those observations to old mice, by comparing the effect of ActR-Fc-nLG3 administration to a myostatin inhibitor alone (ActR-Fc) and to the control vehicle (PBS). We found that the long-term administration of the new protein enhanced motor performance, particularly endurance, in a sustained way. In old mice, this compound has the potential of maintaining the innervation of the hypertrophic muscle, by acting at endplate level. Indeed, we presented indirect evidence that the increased endurance is correlated with the effect of ActR-Fc-nLG3 compound on the NMJs, which show an increase in surface receptor area.

## MATERIALS AND METHODS

### Animals

For the following experiments, we employed 24 male mice, strain C57BL/6J (Charles River, France), age 22 months. Mice were kept in regular cages, 5 per cage, under 12/12-h light/dark cycle, with food and water available *ad libitum*. Ears were punched for identification at the beginning of the experiment. All procedures involving the use of laboratory animals were performed in accordance with the Italian National (DL n. 116, G.U., Supp. 40, February 18, 1992; permit number 17/2010-B, June 30, 2010) and European Communities Council Directive 24 November 1986 (86/609/EEC). The experiments were also approved by the Italian Ministry of Health and the Bioethical Committee of the University of Turin (authorization number 17/2010-B, June 30, 2010). All experiments were designed to minimize the number of animals used and possible discomfort.

### Compounds

All compounds were produced and purified by Evitria AG (Schlieren, Zurich, Switzerland). The ActR-Fc protein (Ramatercept) has been described before [24]. Briefly, it consists of the mouse extracellular part of the ActR-IIB receptor coupled the Fc part of an Igg1 mAb. The ActR-Fc-nLG3 compound was described firstly in Boido et al., 2020 [23]. It consists of the neuronal laminin G3 domain of the neuronal form of agrin [25, 26] -LG3- coupled to the c-terminus of the Fc part of an Igg1 mAb and to the myostatin inhibitor ActR-IIB receptor.

### Treatments

**Figure 1.**
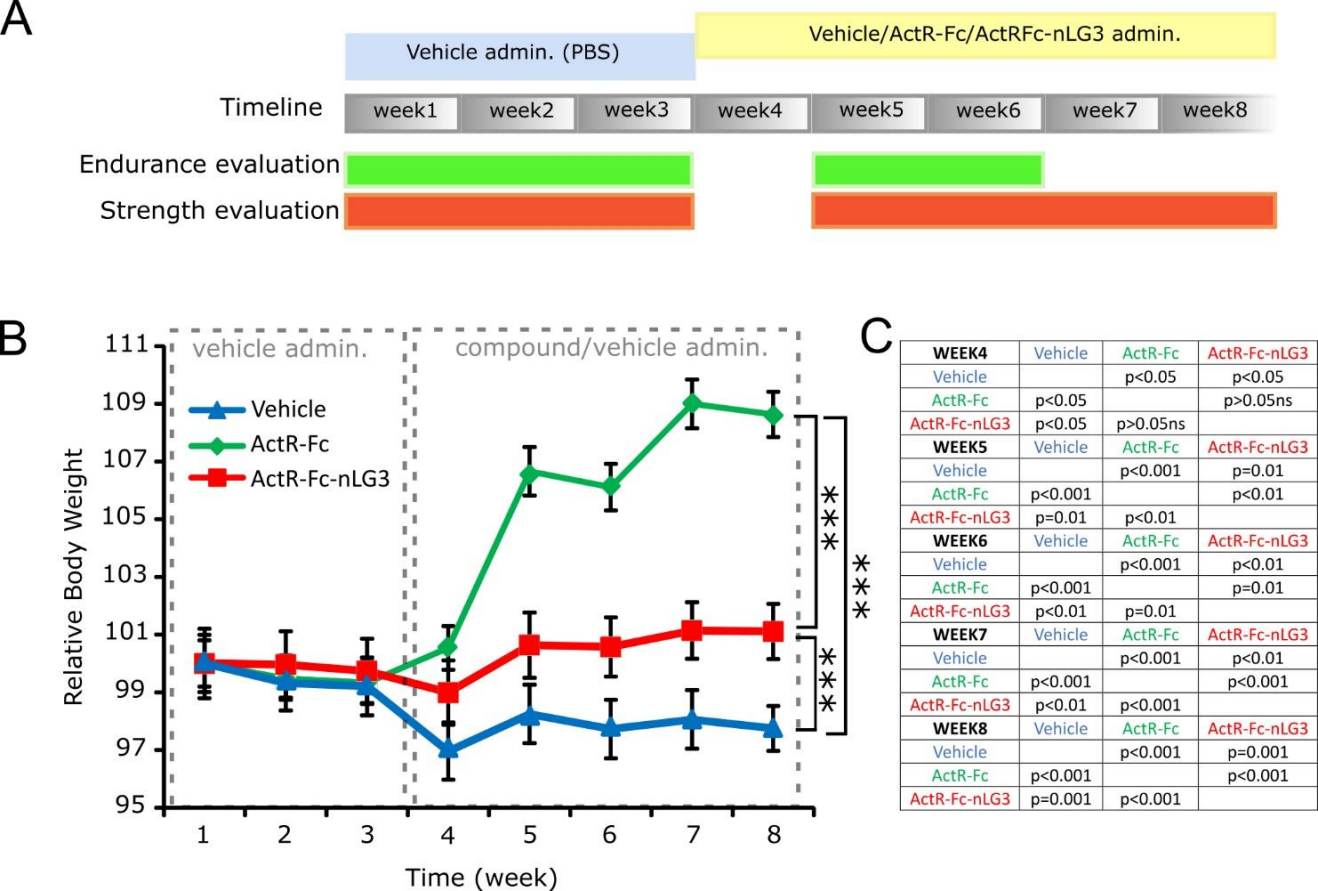
**Experimental procedures and body weight measurement in old mice**. **(A)** Timeline illustrating the experimental procedures. 22-month-old mice were injected for 3 weeks with vehicle (PBS), then randomly divided into the three experimental groups, and injected with vehicle, ActR-Fc or ActR-fc-nLG3 compounds for 5 weeks. Endurance was evaluated in the first 3 weeks and then in week 5 and 6; strength evaluation was performed every week except for week 4. **(B)** Measurement of the relative body weight of mice (vehicle, ActR-Fc and ActR-fc-nLG3 groups) before and after vehicle/compound administration. Two-ways ANOVA, Groups, F_(2,168)_= 65.32; p<0.001; Time, F_(7,168)_= 7.35; p<0.001; Interaction, F_(14,168)_= 6.861; p<0.001. Asterisks indicate the major statistical differences between the three groups. **(C)** Tuckey’s multiple comparisons test values between groups for each time point during treatment administration (weeks 4-8). Data are shown as mean ± s.e.m. N=8 animals per group.

The experiment was conducted for 8 weeks. The animal weight was checked daily. Animals were randomly assigned to one of three groups. During the first three weeks, PBS (vehicle) was administered by subcutaneous (s.c.) injections to all the animals to set baseline condition, then ActR-Fc (10mg/kg), ActR-Fc-nLG3 (10mg/kg), both dissolved in PBS, or the vehicle only were s.c. administered for the last five consecutive weeks, based on the belonging group [23, 27]. Finally, at the end of week 8, mice received the last injection and were sacrificed 24h later (Fig. 1A).

### Treadmill Exercise

To assess motor endurance, mice (n= 24; 8 per group) were trained on the treadmill apparatus (Panlab, Harvard Apparatus) three times per week. Mice underwent an accelerating treadmill protocol for 3 weeks before starting compound administrations, then for 2 weeks (week 5 and 6) during compound/vehicle dosing [27]. Briefly, mice were properly acclimated to the treadmill prior to the experimental sessions. In the days before the experimental runs, mice were placed on the belt of the treadmill in their respective lanes with shocking grids off, and they were let to explore the instrument for minutes. During experimentation, mice were warmed up for 2 minutes before running: warm-up procedure consists in starting the belt at a slow speed (16 cm/sec) and then slowly turning on the shocking grids to 0.2 mA. After the warm-up period, mice were tested for their running performances. The treadmill speed started from 16 cm/sec and then accelerated by 1cm every minute; the acceleration continued until the mouse reached the exhaustion state, that we set up as the moment when a mouse receives 10 or more-foot shocks in one minute [28], at which point the experiment was stopped. After exhaustion, the shocking grid was deactivated, and the mouse was returned to its cage. For each treatment group, the running distance (m) and the number of foot shocks (total and per minute) experienced were recorded.

### Grip Strength Assessment

Forelimb grip strength was measured using a Grip Strength Meter (Ugo Basile, Varese, Italy). Control and treated mice (n= 8 each group) were tested twice a week during the first three weeks of the experiments and in week 5 and 6, then were tested five times a week in the last two weeks of experimentation (w7-8). Mice were held by the tail and allowed to grasp a T-shaped bar with their forepaws. Once the mouse grasped the bar with both paws, it was pulled away from the bar until releasing the bar. The apparatus displayed the level of tension (gram force) exerted on the bar by the mouse. Each animal was given five consecutive tests during the day; the lowest and the highest values were excluded from the analysis, and the average value was recorded.

### Animal sacrifice, Muscle Isolation and Sectioning

24h after the last treatment dosing, mice (n=23) were sacrificed by cervical dislocation. Fresh triceps, gastrocnemius and quadriceps skeletal muscles were quickly dissected out from the skin and bones by forceps and scissors. The wet muscle weight was determined immediately after isolation. Then, the muscles were embedded in OCT medium (Bio-Optica, Milan; 05-9801), and rapidly frozen in cold isopentane (2-Methylbutane; Sigma-Aldrich; M32631) for cryosectioning (Leica). For long-term storage, samples were kept at -80°C.

20 (cross) and 25 (parasagittal) μm-thick muscle sections were cut on the cryostat (Leica) and collected on 4% gelatin-coated slides (ThermoScientific Menzel Gläser, 217655). The sections were dried at RT for 1 hour and then stored at -20°C.

### Hematoxylin/Eosin staining, Immunohistochemistry and Morphometrical Analysis

To morphologically analyse the muscles (cross-sectional area, number of myonuclei per fiber), triceps sections (ActR-Fc, n=7; ActR-Fc-nLG3, n=8; vehicle, n=8) were stained with Hematoxylin and Eosin Y 1% aqueous solution (Bio-Optica). Muscle sections were then dehydrated in ascending series of ethanol (95-100%; Sigma-Aldrich) and cleared in xylene (98.5%; Carl Roth).

Hematoxylin/Eosin-stained muscles were analysed with a Nikon Eclipse 90i microscope and photographed by a Nikon DS-5Mc digital camera, using a 20x magnification objective. Three pictures were taken for each animal. In every picture, the number of peripheral nuclei, and the number of fibers, were quantified using ImageJ software (MIT). The cross-section area, perimeter and Feret diameters of each muscular fiber were measured using ImageJ software.

For the analysis of laminin expression and muscle myosin heavy chains (MHC), after being rinsed in PBS, triceps cross sections (n=4 animals for each group) were incubated for 24h at 4°C with the following primary antibodies, diluted in 0.1 M PBS, pH 7.4, 0.5% Triton X-100, and appropriate 2% normal sera: anti-laminin (#L9393; Merck Millipore; 1:400); anti-myosin, slow (monoclonal; #M8421, Merck Millipore; 1:400); anti-myosin, fast (monoclonal; #M4276, Merck Millipore; 1:400). The following day, proper secondary antibodies (AlexaFluor-488 anti-rabbit IgG, #711-165-152; and Cyanine-Cy3 anti-mouse IgG, #715-165-150; Jackson ImmunoResearch Laboratories, West Grove, PA, USA) were used 1:300 in PBS for 1.30 h at RT, followed by DAPI incubation (#D1306; Thermo Fisher; 1:100) in PBS. Sections were then coverslipped with the anti-fade mounting medium Mowiol (Sigma-Aldrich). For each animal, 200 muscle fibers were drawn by Neurolucida software (MicroBrightField Inc, Williston, VT, USA), and the number of DAPI+ nuclei per fiber, and the percentage of MHC-expressing fibers were counted. Representative images of MHC fast and slow- expressing fibers were acquired (step size 0.69μm, magnification 40x with zoom 1.8, acquisition speed 100 Hz, format 1024 x 1024 pixels) using a Leica TCS SP5 confocal laser scanning microscope (Leica Microsystems, Wetzlar, Germany).

For the analysis of NMJs, 25μm-thick parasagittal gastrocnemius muscle slices were incubated for 30 min at room temperature with the Alexafluor-555-conjugated bungarotoxin (BTX; #B35451; Invitrogen), diluted 1:1000 in 0.1 M PBS, pH 7.4, 0.5% Triton X-100. Then, for immunofluorescence staining, the sections were incubated in a mixture of primary antibodies diluted in 0.1 M PBS, pH 7.4, 0.5% Triton X-100, and appropriate 2% normal sera for 24h at 4°C. The following primary antibodies were used: anti-neurofilament protein (NF-M, 145kDa; #MAB1621, Millipore; 1:500); anti phospho-MuSK (Tyr755) (#PA5-105382; Invitrogen; 1:100); and ACVR-2b (Invitrogen; 1:100). Next, proper secondary antibodies (AlexaFluor-488 anti-mouse IgG, #715-165-150, and AlexaFluor-647 anti-rabbit IgG, #711-605-152; Jackson Immuno Research Laboratories, West Grove, PA, USA) were used 1:300 in PBS for 1.30 h at RT, followed by 3 washes with PBS and 3 min incubation at RT with DAPI (Thermo Fisher; 1:100) in PBS. Finally, sections were coverslipped with the anti-fade mounting medium Mowiol. Slices were analysed with a Leica TCS SP5 confocal laser scanning microscope (Leica Microsystems).

Secondary antibody-only controls were used on muscle slices to validate antibody specificity and distinguish the immunopositivity staining from background.

To scan the entire NMJs, confocal stacks (22 serial sections, step size 0.69μm, magnification 63x with zoom 3.0x, acquisition speed 100 Hz, format 1024 x 1024 pixels) were acquired using a Leica TCS SP5 confocal laser scanning microscope (Leica Microsystems).

NMJs were considered as fragmented when synaptic 'spots' of BTX and fractured NF signals were observed in the endplates, rather than a continuous extension of BTX+ ‘pretzel-like' NMJ structure, together with entire and intact NF+ nerve fibers (innervated NMJ). NMJs were considered denervated when the NF+ fibers were not contacting BTX+ endplates. To have a standardized approach to the morphometric analysis of the NMJ, about 70 NMJ images for each experimental group, acquired by confocal microscopy (as mentioned above), were analysed using the α-NMJ Macro ImageJ plugin, specially designed for the rapid automated analysis of endplate morphology and pre-synaptic branches [29]. Feret diameters (maximum and minimum Feret) of the NMJs of 3 animals per group (10 NMJs per animal) were analysed using ImageJ software.

For the analysis of NFs and p-MuSK or ACVR-2b expression, confocal stacks were analysed using Imaris software (Bitplane): for each animal, at least 10 entire, not fragmented, NMJs were imaged and reconstructed in 3D by Imaris software; thus, the % of NF, p-MuSK or ACVR-2b signal accumulation in NMJ volume was evaluated by calculating the ratio between marker labelling volume and α-BTX (in NMJ) labelling volume.

Images were used for analysis or assembled into extended focus photographs, with brightness, colour, and contrast balanced using ImageJ, and assembled into panels with Inkscape (Free vector graphics editors). The histological images included in the figures provide an accurate representation of the average outcomes derived from our data analyses, to enhance the overall impact and comprehensibility of the research findings.

### Statistical Analysis

Data are shown as means ± standard error of mean (s.e.m.). Statistical analyses were performed by GraphPad Prism 8.0 software (GraphPad Software, San Diego, CA, USA). We first tested the normal distribution of the data with Kolmogorov-Smirnov and Shapiro-Wilk tests of normality.

In the behavioral analysis, the data of mice weight, running endurance and grip strength in time were analysed using two-ways ANOVA, followed by the Tuckey’s multiple comparisons as a post-hoc test. Using analysis of variance tests, the between-subjects factor of Group and within-subjects factor of Time were used.

Moreover, one-way ANOVA followed by Tuckey’s multiple comparisons as a post-hoc test was performed before and after treatment periods in the analysis of the endurance and grip strength. Paired t-test was performed within each single group to compare before and after treatment data.

In the analysis of foot shocks, to obtain an estimate of the performance before and after treatment, we performed a linear regression on pulses per minute values for each experimental group.

In the histological analysis, one-way ANOVA followed by the Tuckey’s multiple comparisons test was performed for the statistical analysis of muscle wet weight, fiber CSA, number of nuclei, percentage of MHC-expressing fibers, and in the analysis of NMJ dimensions and marker intensity expression. One-way ANOVA followed by Tuckey’s multiple comparisons test was also used to analyse the data generated from aNMJ-morph macro, for NMJ parameters comparisons between groups. The analyses were performed blinded for the treatment of the mice. Results were considered to be statistically significant when p was <0.05 *; p<0.01 **; p<0.001 ***.

**Table 1:** Summary of the main results obtained in this study.

	compared to the pre-treatment period				compared to vehicle-treated animals				
	Body Weight	Endurance	Foot shocks	Strength	Muscle weight	Myonuclei	NMJs innervation	NMJs dimensions	p-Musk signal
Vehicle	↓	↓↓	↑	↓					
ActR-Fc	↑↑	↓	↑	↑	↑↑	↑↑	=	=	=
ActR-Fc-nLG3	↑	↑↑	↓	↑	↑	=	↑↑	↑	↑↑

↑, significant increase; ↑↑, very significant increase; ↓, significant decrease; ↓↓, very significant decrease; =, no changes.

## RESULTS

All the animals tolerated the treatments very well. In particular the ActR-Fc-nLG3 and control animals did not show adverse events nor behavioral or physical abnormalities. On week 8, we observed a single death in the ActR-Fc group which was believed to be associated with old age (> 22 months).

All the results obtained in this study are summarized in Table 1.

### ActR-Fc-nLG3 increases mice weight compared to vehicle

ActR-Fc and ActR-Fc-nLG3 compounds were chronically administered to old mice for five consecutive weeks, starting from week 4, while control group received PBS (Fig. 1A). Starting from the first experimental week (w1), the animal weight was daily recorded. While in the first weeks of the experimental procedure (weeks 1-3, PBS administration) no differences in weight were visible among the three groups, during the treatment period (w4 to 8) we found that peptide administration significantly increased relative body weight in both ActR-Fc and ActR-Fc-nLG3 mice-treated groups, compared to controls (two-ways ANOVA, Groups, p<0.001; Time, p<0.001; Interaction, p<0.001. Fig. 1B, 1C). Interestingly, the administration of ActR-Fc peptide resulted in the highest increase in body weight from the beginning to the end of treatment: indeed, the increase in average body weight was 1.47% for vehicle, 11.0% for Actr-Fc and 5.07% for ActR-Fc-nLG3 groups.

**Figure 2.**
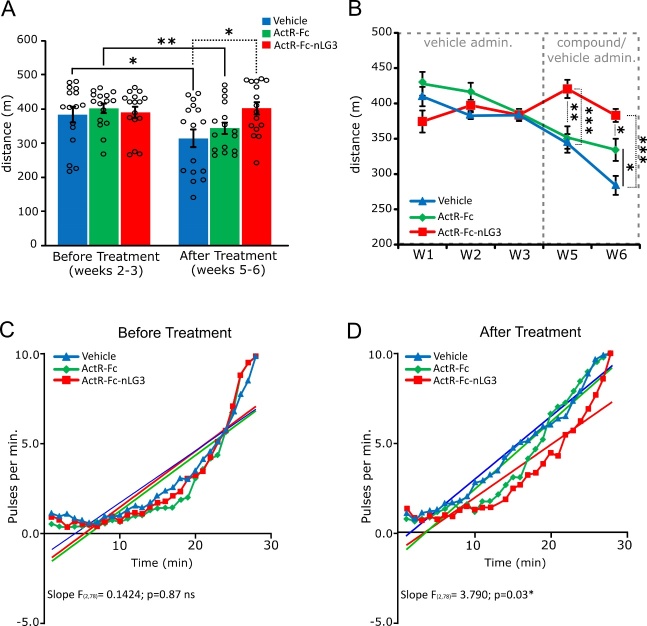
**ActR-Fc-nLG3-treated old mice show increased endurance in treadmill exercise**. **(A)** Measure of the average distance run by the mice on the treadmill before and after vehicle/compound administration. Both vehicle and ActR-Fc groups show the worsening of running performances after treatment, while no changes were observed for ActR-Fc-nLG3 group. One-way ANOVA, F_(2,45)_= 4.408; p<0.05. Paired t-test before treatment Vs after treatment, *p<0.05; **p<0.01; ***p<0.001. N= 2 measurements per animal; 8 animals per group. **(B)** Distance run by the 3 experimental groups for every experimental week, before and after vehicle/compound treatment. ANOVA for repeated measure, Groups, F_(2;101)_= 8.698; p=0.0003; Time, F_(4,101)_= 15.33, p<0.001; Interaction, F_(8,101)_= 6.276; p<0.001, followed by Tuckey’s multiple comparisons test (*p<0.05; **p<0.01; ***p<0.001). N=8 animals per group. **(C-D)** Average number of foot shocks per minute received by the mice during treadmill test before (C) and after (D) the treatment with the respective linear regressions and Slope value. ActR-Fc-nLG3 treated mice received a lower number of foot shocks compared to the other 2 groups. Every data point is the average of 48 experimental data (N= 8 animals per group, 6 runs). Data are shown as mean ± s.e.m.

### ActR-Fc-nLG3-treated old mice showed increased endurance in treadmill exercise

Controls and treated old mice were tested for endurance on the treadmill before (in weeks 1 to 3, but endurance was calculated starting from w2) and after (w5-6) treatment. In the training period, before treatment (computed as the mean values of weeks 2 and 3), no differences were observed in the distance run between controls and the treated groups (vehicle 383m ± 14.1; ActR-Fc 401.3m ± 8.9; ActR-Fc-nLG3 390.4m ± 10.1. One-way ANOVA; p>0.05 ns), but differences between groups in the running distance were observed after treatment (vehicle 313.6m ± 15.7; ActR-Fc 343m ± 11.3; ActR-Fc-nLG3 401.8m ± 11.7. One-way ANOVA p=0.018) (Fig. 2A). Interestingly, after three dosing weeks, comparing the before and after treatment performances, both the vehicle and the ActR-Fc treated animals showed a statistically significant decrease in running distance (vehicle, p=0.011; ActR-Fc, p=0.0013), while ActR-Fc-nLG3-treated mice showed no difference in running performance (p=0.64 ns). Indeed, after the treatment weeks, the average distance run decreased of 18.27% in vehicle-treated animals and of 14.46% in ActR-Fc group, while was increased of 2.82% in ActR-Fc-nLG3 treated mice (Fig. 2A). Thus, the motor performance of ActR-Fc-nLG3-treated mice was moderately increased notwithstanding the significantly older age, suggesting that this peptide supported the endurance capacity in running exercise in these old animals. The effect of ActR-Fc-nLG3 peptide was visible soon after the first week of administration. Indeed, by looking at the running distance over time (from w1 to w6), significant differences were observed from week 5 in ActR-Fc-nLG3 group compared to vehicle and to ActR-Fc groups (ANOVA for repeated measures; Groups, p=0.0003; Time, p<0.001; Interaction, p<0.001; Fig. 2B). This result was supported by the data on the number of experienced shocks. In the first three weeks of the experiment, during vehicle administration, all the three groups showed a similar behaviour, as they experienced a similar number of foot shocks (linear regression, p=0.87 ns.) (Fig. 2C). Instead, during the dosing period, by measuring the total number of pulses per minute received by the animals, we found that in the first part of the running exercise (i.e., first 15 minutes) ActR-Fc and ActR-Fc-nLG3-treated animals experienced a similar number of foot shocks. However, the performance of ActR-Fc-injected animals gradually worsened (increased number of pulses) compared to the ActR-Fc-nLG3 group, resulting in a shorter running distance (Fig. 2D).

Thus, ActR-Fc-nLG3 administration seems to support better motor performance, in comparison to ActR-Fc. The vehicle group registered the worse results, since animals received the highest number of shocks immediately after the beginning of the exercise (linear regression, p=0.0269) (Fig. 2D). These results support the hypothesis that ActR-Fc-nLG3 administration can sustain the endurance in old mice, leading to better motor performance.

**Figure 3.**
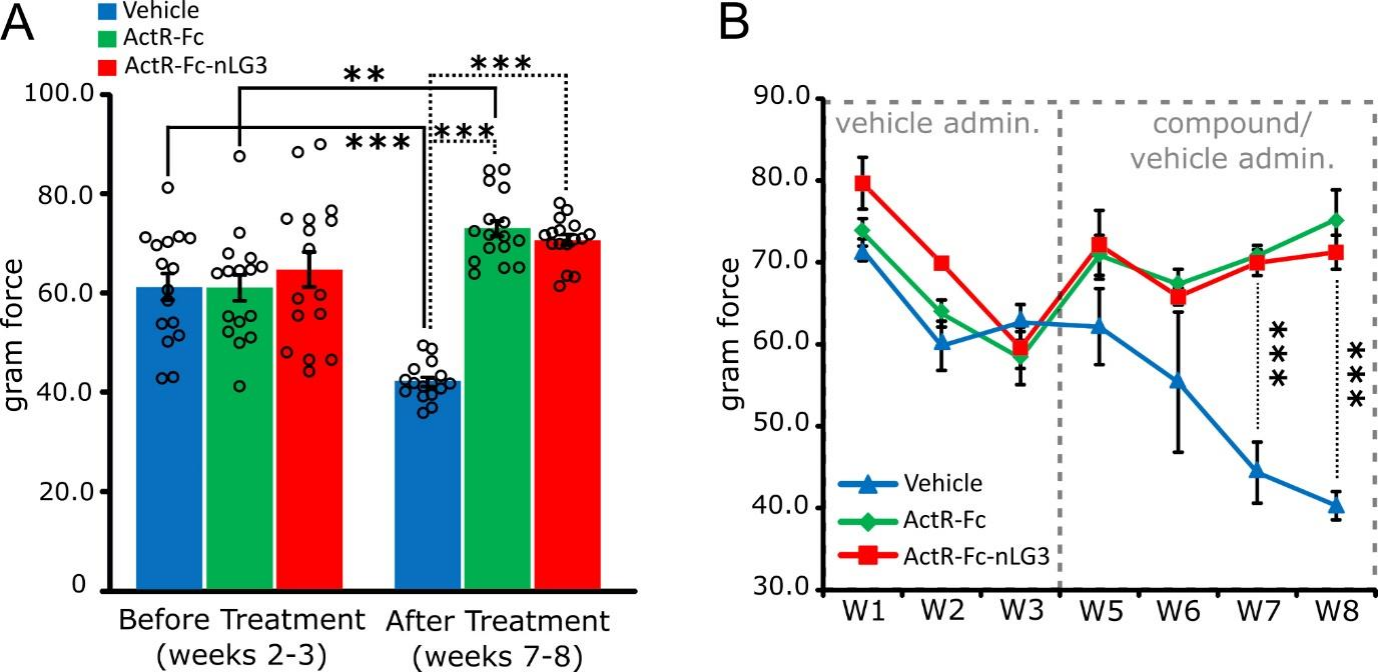
**Grip strength performance is increased after ActR-Fc-nLG3 administration**. **(A)** Mean gram force recorded in the grip strength test for the 3 experimental groups before and after vehicle/compound treatment. An increase in strength was observed after treatment in ActR-Fc and ActR-Fc-nLG3 groups, while a decrease was seen in control group. One-way ANOVA before treatments, F_(2,45)_= 0.4478; p>0.05 ns. After treatments, F_(2,45)_= 164.9; p<0.001. Paired t-test before treatment Vs after treatment; *p<0.05; **p<0.01; ***p<0.001. N= 2 measurements per animal; 8 animals per group. **(B)** Gram force for each experimental group recorded every week. ANOVA for repeated measures; Groups, F_(2;147)_= 37.20, p<0.001; Time, F_(6;147)_= 7.860, p<0.001; Interaction, F_(12;147)_= 5.704, p<0.001; followed by Tuckey’s multiple comparisons test (*p<0.05; **p<0.01; ***p<0.001). N= 8 animals per group. Data are shown as mean ± s.e.m.

### Grip strength performance is increased after ActR-Fc-nLG3 administration

The increase in weight and running endurance observed in ActR-Fc-nLG3 animals could suggest an improvement in strength in these mice. Thus, we performed a grip strength test to assess mice performance before and during treatment. As a consequence of the positive results obtained in treadmill exercise in ActR-Fc-nLG3 treated group (w5-6), we decided to extend the window for strength assessment by two weeks, considering the mean values of weeks 7 and 8 for final strength measurement in the treatment period. No differences in strength were observed in the weeks before treatment (w2-3) (one-way ANOVA; p=0.64 ns). Conversely, during the treatment period we observed a significant increase in strength both in ActR-Fc- and AcrR-Fc-nLG3 treated mice, compared to vehicle-treated animals (one-way ANOVA; p<0.001; Fig. 3A). Comparing the performances before and after treatment, we found an increase in gram force in the ActR-Fc and AcrR-Fc-nLG3 animals, although the latter was not statistically significant (p=0.07; Fig. 3A). Conversely, the vehicle group showed a significant reduction in strength in the last weeks of the experiment compared to the initial phase (p<0.001; Fig. 3A), indicating a progressive weakening in the untreated old animals.

Indeed, by looking at the time course of the force improvement (from w1 to w8), the effect of the two compounds was visible since the beginning of the treatment, with statistically significant differences (p<0.001) in comparison with vehicle group at weeks 7 and 8 (Fig. 3B). Thus, the administration of ActR-Fc or AcrR-Fc-nLG3 compounds increased mice strength compared to control condition, reflecting the increase in weight observed in both treated groups and the improvement in motor performance found in the ActR-Fc-nLG3 group.

**Figure 4.**
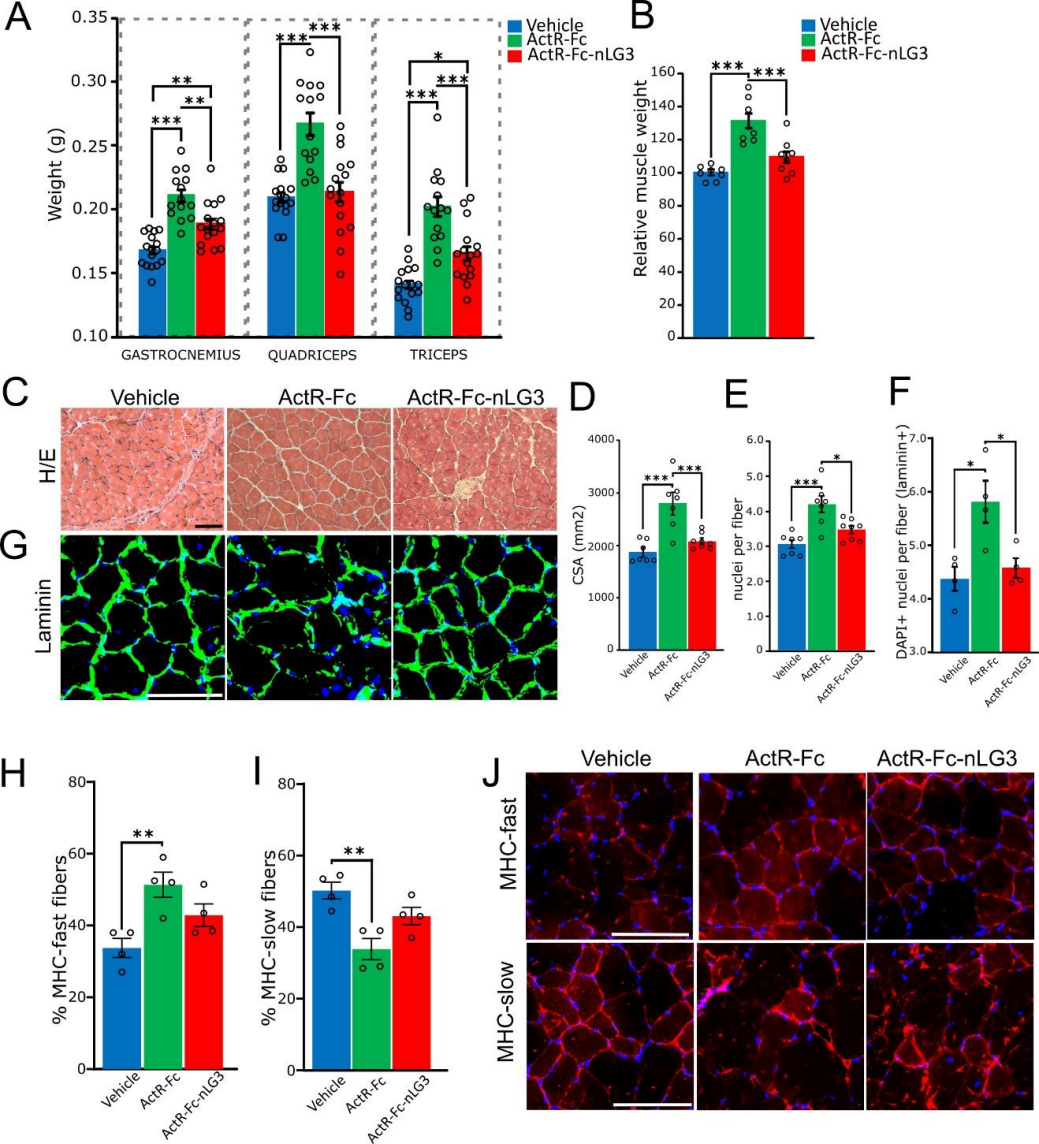
**The anti-myostatin induced hypertrophy is quenched by ActR-Fc-nLG3, and no major histological changes are observed in ActR-Fc-nLG3 treated muscle fibers compared to controls**. **(A)** Measurement of freshly dissected gastrocnemius, quadriceps and triceps muscle weight. ActR-Fc group shows the highest values of muscle weight. Gastrocnemius; one-way ANOVA, F_(2;43)_= 26.00; p<0.0001; Triceps; one-way ANOVA, F_(2;43)_= 27.52; p<0.0001; Quadriceps; one-way ANOVA, F_(2;43)_= 18.96; p<0.0001; followed by Tuckey’s multiple comparisons test (*p<0.05; **p<0.01; ***p<0.001). N= 2 muscles per animal; 8 mice, vehicle; 7, ActR-Fc; 8, ActR-Fc-nLG3. **(B)** Relative increase in muscle weight of ActR-Fc and ActR-Fc-nLG3 groups compared to vehicle group, used as reference value. One-way ANOVA, F_(2;135)_=28.72; p<0.001; followed by Tuckey’s multiple comparisons test (*p<0.05; **p<0.01; ***p<0.001). N= 8, vehicle; 7, ActR-Fc; 8, ActR-Fc-nLG3. **(C)** Hematoxylin/eosin (H/E)-stained representative images showing vehicle, ActR-Fc and ActR-Fc-nLG3 triceps fibers and myonuclei. **(D)** Cross-section area (CSA) measurement showing the increase in fiber dimensions in ActR-Fc group. One-way ANOVA, F_(2,19)_= 17.18; p<0.001 followed by Tuckey’s multiple comparisons test (*p<0.05; **p<0.01; ***p<0.001). N= 8, vehicle; 7, ActR-Fc; 8, ActR-Fc-nLG3; 100 fibers each animal. **(E-F)** Number of myonuclei per fiber in the three experimental groups evaluated in H/E-stained tissue (E) and in laminin-stained muscle fibers (F). ActR-Fc-treated animals show a significantly higher number of myonuclei in both the analysis. H/E staining, one-way ANOVA, F_(2,19)_=12.76; p<0.001; followed by Tuckey’s multiple comparisons test (*p<0.05; **p<0.01; ***p<0.001). N= 8, vehicle; 7, ActR-Fc; 8, ActR-Fc-nLG3; 100 fibers each animal. Laminin-DAPI staining, one-way ANOVA, F_(2,9)_=7.803; p<0.05; followed by Tuckey’s multiple comparisons test (*p<0.05; **p<0.01; ***p<0.001). N= 4 animals per group, 200 fibers each animal. **(G)** Representative images showing laminin staining in the triceps fibers of the 3 experimental conditions. Myonuclei are labelled with DAPI. **(H-I)** Percentage of myosin heavy chains (MHC) fast (H) and slow (I) -expressing fibers in the three experimental groups. MHC-fast; one-way ANOVA, F_(2,9)_=7.988; p<0.05; MHC-slow; one-way ANOVA, F_(2,9)_=10.02; p<0.01; followed by Tuckey’s multiple comparisons test (*p<0.05; **p<0.01; ***p<0.001). N= 4 animals per group, 200 fibers each animal. **(J)** Representative images of MHC fast (upper panels) and slow (lower panels) expression in muscle fibers in the three experimental conditions. Scale bar: 200 µm (C); 100 µm (G, J). Data are shown as mean ± s.e.m.

### Muscle weight is increased after ActR-Fc-nLG3 chronic treatment

To correlate the body weight trend observed in ActR-Fc and ActR-Fc-nLG3-treated mice to a possible muscle size increase, mice were then sacrificed and gastrocnemius, quadriceps, triceps muscles dissected. The wet muscle weights were determined immediately after the isolation. We found that both compounds significantly increased the weight of gastrocnemius (one-way ANOVA; p<0.0001) and triceps (p<0.0001) muscles, compared to controls (Fig. 4A). A significant increase in quadriceps weight was observed only in ActR-Fc group (one-way ANOVA; p<0.0001), suggesting that the myostatin inhibitor ActR-Fc played a major effect on muscle trophism (Fig. 4A). Indeed, the administration of the ActR-Fc and of ActR-Fc-nLG3 respectively induced a 31% and almost 10% increase of muscle wet weight, compared to PBS-treated animals (Fig. 4B). Thus, the overall body weight increase observed in both ActR-Fc and ActR-Fc-nLG3-treated animals can be related to an augmented muscle mass.

### ActR-Fc-nLG3 old animals show signs of reduced muscle atrophy compared to ActR-Fc group

In our analysis, ActR-Fc and ActR-Fc-nLG3 groups showed an increase in both body and muscle weight after treatment. Moreover, only the ActrR-Fc-nLG3-treated mice were able to maintain good motor performance in the treadmill exercise suggesting that this peptide can sustain endurance.

To correlate motor performance with possible histological changes, we examined and measured hematoxylin-eosin-stained fibers of triceps muscles (Fig. 4C). We selected this hindlimb muscle because of the differences in fresh muscle weight we observed among all the groups. We found that muscle fiber area was significantly increased (one-way ANOVA, p<0.001) in ActR-Fc-treated animals compared to ActR-Fc-nLG3 and control groups (Fig. 4D).

Subsequently, we counted the number of myonuclei per fiber in H/E-stained slices and found an increase in ActR-Fc animals compared to ActR-Fc-nLG3-treated group and controls (one-way ANOVA; p<0.001; Fig. 4E). We confirmed the increase in peripheral nuclei in ActR-Fc group by counting DAPI+ nuclei in laminin-stained muscle fibers (one-way ANOVA, p<0.05; Fig. 4F). In this latter analysis, we also observed no differences in nuclei number between vehicle an ActR-Fc-nLG3 treated groups (Tuckey’s multiple comparisons test, p>0.05) (Fig. 4F-G).

In the same tissue (triceps muscle) we then looked at the expression of fast and slow myosin heavy chains (MHCs) in fibers (Fig. 4H-J). Interestingly, ActR-Fc treated muscles showed an increase in the percentage of MHC-fast fibers compared to vehicle (one-way ANOVA; p= 0.01; Tuckey’s multiple comparisons test; vehicle vs ActR-Fc, p<0.01) (Fig. 4H), and a decrease in the percentage of MHC-slow fibers (one-way ANOVA; p=0.005; Tuckey’s multiple comparisons test; Vehicle vs ActR-Fc, p<0.01) (Fig. 4I), suggesting a switch from oxidative to glycolytic metabolism in the muscle fibers after myostatin inhibition. ActR-Fc-nLG3 treatment showed a more modest effect on MHC types: indeed, compared to vehicle, a slight increase in MHC-fast, and a decrease in MHC-slow fiber percentage were observed, without reaching statistical significance (p>0.05) (Fig. 4H-J). Thus, data suggest a more oxidative phenotype in fibers treated with ACtR-Fc-nLG3 compared to fibers treated with ActR-Fc.

**Figure 5.**
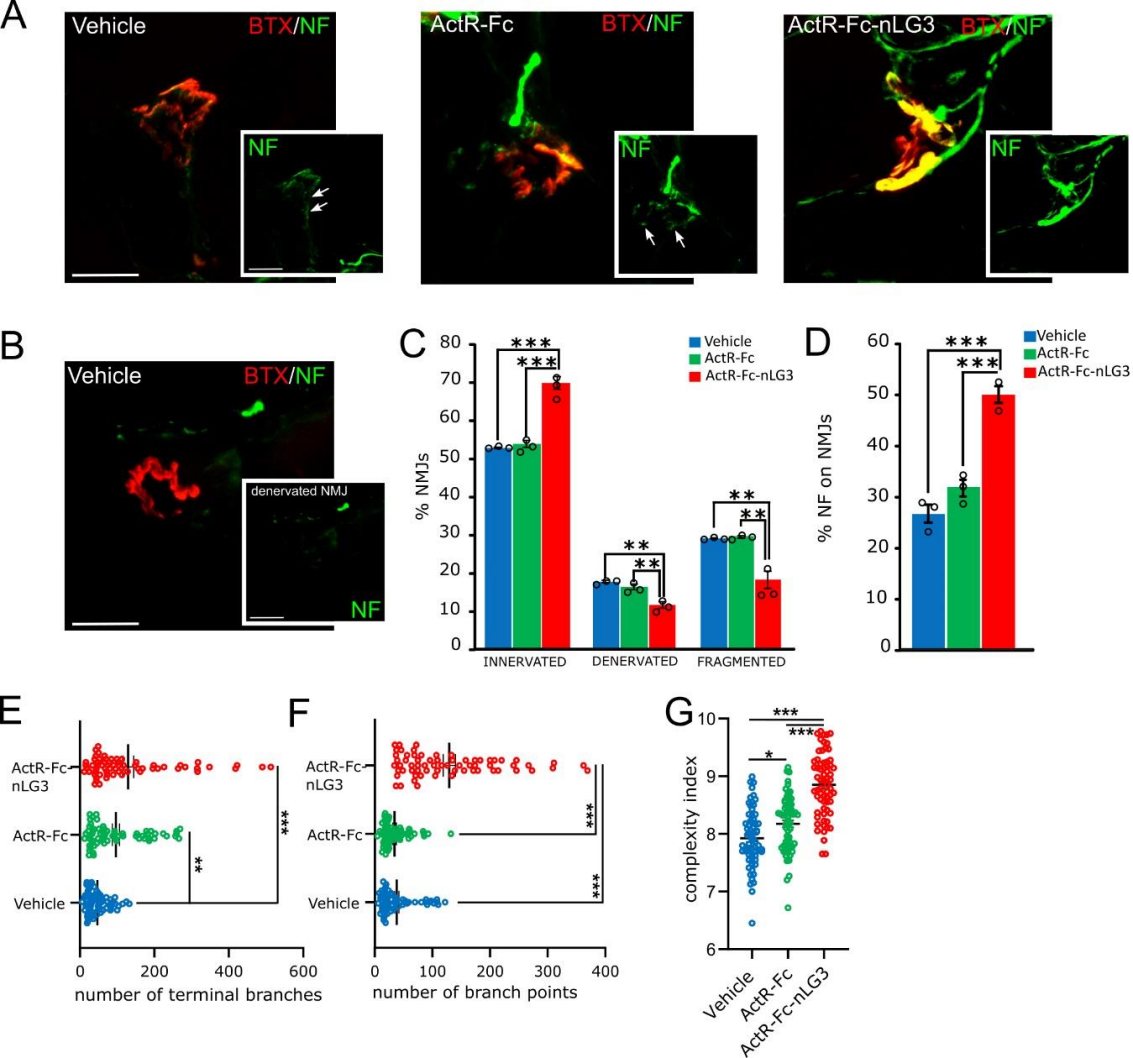
**Innervation is preserved in ActR-Fc-nLG3 treated NMJs**. **(A)** Representative confocal images of NMJs labelled by α-Bungarotoxin (BTX, in red) of the 3 experimental groups showing the increase of NF signal (in green) in ActR-Fc-nLG3 endplates. The lowest NF+ signal is observed in NMJ treated with vehicle (PBS). White arrows indicate fractured NF inside NMJs of vehicle and ActR-Fc treated groups. **(B)** Representative image of a denervated NMJ in vehicle group. No NF+ green filaments are observed inside BTX+ endplate. **(C)** Quantification of the percentage of innervated, denervated and fragmented NMJs showing the improvement in innervation in ActR-Fc-nLG3 group. Innervated NMJs, one-way ANOVA, F_(2,6)_=75.67, p<0.001; denervated NMJs; one-way ANOVA, F_(2,6)_=23.66, p<0.01; fragmented NMJs; one-way ANOVA, F_(2,6)_=23.44, p<0.01; followed by Tuckey’s multiple comparisons test (*p<0.05; **p<0.01; ***p<0.001). N=3 animals each group, 10 NMJs each animal. **(D)** Quantification of the percentage of NF signal volume still present within the endplate (NMJ volume) in all the experimental groups, showing the highest percentage values in ActR-Fc-nLG3 treated NMJs. One-way ANOVA, F_(2,6)_= 51.3; p<0.001 followed by Tuckey’s multiple comparisons test (*p<0.05; **p<0.01; ***p<0.001). N=3 animals each group, 10 NMJs each animal. (E-F-G) Analysis of the number of terminal branches (E), branch points (F) and complexity index (G) with aNMJ-morph macro (ImageJ). ActR-Fc-nLG3 NMJs show the highest number of terminal branches and branch points, together with the highest complexity index. Terminal branches; one-way ANOVA, F_(2,194)_=16.22, p<0.001; number of branch points; one-way ANOVA, F_(2,194)_= 64.88, p<0.001; complexity index, one-way ANOVA, F_(2,194)_= 59.09, p<0.001; followed by Tuckey’s multiple comparisons test (*p<0.05; **p<0.01; ***p<0.001). N= 4 animals per group, ≈ 70 NMJs per animal.

**Figure 6.**
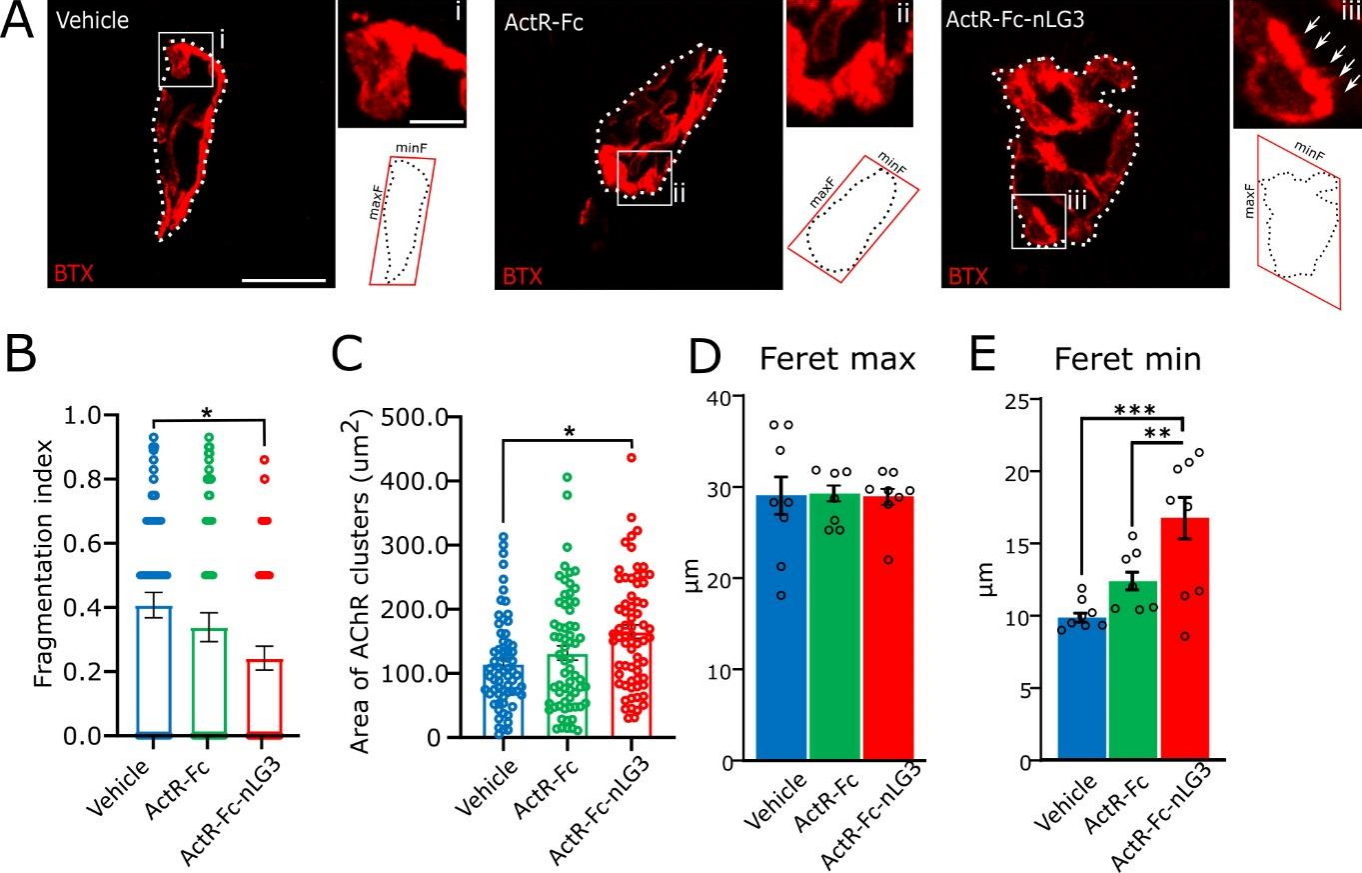
**Amelioration of NMJ dimensions in ActR-Fc-nLG3 group as a sign of preserved innervation**. **(A)** Representative confocal images of NMJs labelled by α-Bungarotoxin (BTX, in red) for binding ACh receptors, showing the morphometrical analysis performed for each experimental group. Insets (i, ii, iii) show a portion of the endplates in higher magnification; amplification of NMJ surface area is observed only in ActR-Fc-nLG3 treated animals (iii; white arrows). **(B-C)** Fragmentation index (B) and area of AChR clusters (C) evaluated with aNMJ-morph macro. Fragmentation index, One-way ANOVA, F_(2,194)_= 4.196, p<0.05; area of ACh clusters, One-way ANOVA, F_(2,194)_= 6.229, p<0.01; followed by Tuckey’s multiple comparisons test (*p<0.05; **p<0.01; ***p<0.001). N= 4 animals per group, ≈ 70 NMJs per animal. Analysis of NMJ Feret max (D) and Feret min (E) diameter. Feret Max, one-way ANOVA, F_(2;20)_=0.014, p>0.05 ns. Feret Min, one-way ANOVA, F_(2;20)_=14.22, p<0.001; followed by Tuckey’s multiple comparisons test (*p<0.05; **p<0.01; ***p<0.001). N= 8 animals, vehicle; 7, ActR-Fc; 8, ActR-Fc-nLG3; 10 NMJs per animal. Scale bars: 10 µm (images in A); 2.5 µm (insets). Abbreviations: minF, minimum Feret diameter; maxF, maximum Feret diameter. Data are shown as mean ± s.e.m.

### Muscular innervation in old animals is preserved after ActR-Fc-nLG3 treatment.

A significant increase in gastrocnemius weight was observed in ActR-Fc-nLG3 and ActR-Fc groups compared to control (Fig. 4A). Thus, to gain morphological insights underpinning motor performance improvement, we also looked at NMJs in gastrocnemius. We evaluated NMJ innervation by looking at the NF fibers contacting the endplate (Fig. 5A). With aging NMJs are subjected to remodelling, showing a reduction in postsynaptic folds and nerve terminal area [30], together with fragmented AChRs and varicose nerve terminals [31] and denervation [32]. We discriminated among NMJs that appeared innervated, denervated or fragmented (see Fig. 5A and 5B for examples). In ActR-Fc-nLG3 treated group we found a higher percentage of innervated junctions (vehicle 53%, ActR-Fc 54%, ActR-Fc-nLG3 69.9%), compensated by a significant decrease (p<0.01) in the percentage of both denervated and fragmented NMJs (Fig. 5C). We quantified the percentage of NF signal volume still present within the endplate (NMJ volume) and observed a significant increase in NF presence in NMJs of mice treated with ActR-Fc-nLG3 than the other two groups (one-way ANOVA; p<0.001; Fig. 5D). To strengthen these observations, we also analysed ≃ 70 NMJs for each group with aNMJ-morph macro [29]. In the analysis of the pre-synaptic side, we found the highest number of terminal branches in ActR-Fc-nLG3 group (one-way ANOVA; p<0.001) (Fig. 5E). Moreover, ActR-Fc-nLG3 treated NMJs exhibited a significant higher number of branch points compared to the other two groups (one-way ANOVA, p<0.001) (Fig. 5F). Finally, ActR-Fc-nLG3 treated NMJs showed the highest complexity index (8.852 ± 0.07), compared to vehicle (7.926 ± 0.06) and ActR-Fc (8.176 ± 0.06) (one-way ANOVA; p<0.001) (Fig. 5G). Thus, it is conceivable that the reduction in denervation observed in the Actr-Fc-nLG3 group leads to the observed improvement in motor performance and strength.

**Figure 7.**
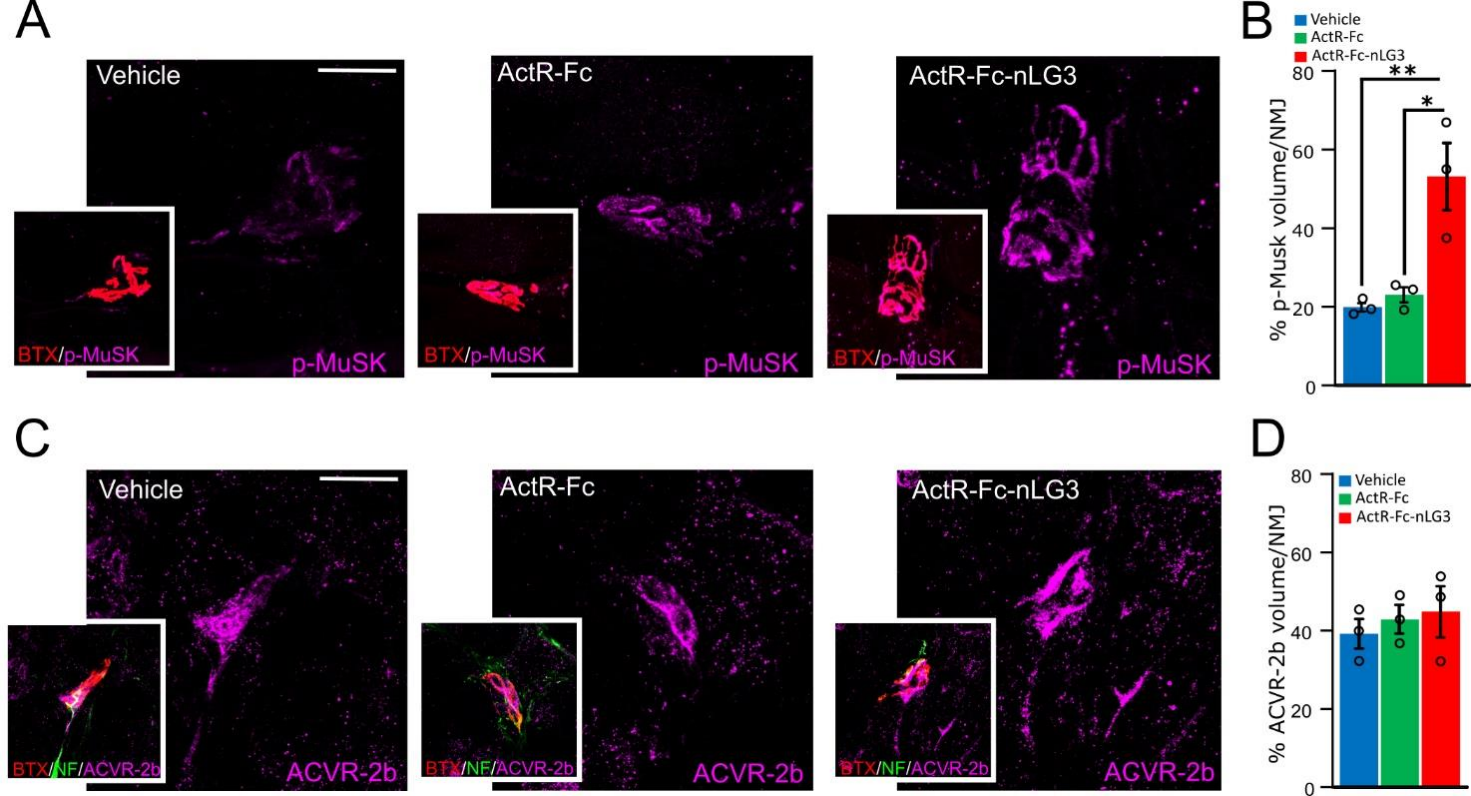
**p-MuSK but not ACVR-2b signaling is increased in the NMJs of ActR-Fc-nLG3 treated mice**. **(A)** Confocal images showing p-MuSK signal intensity in the endplates of vehicle, ActR-Fc and ActR-Fc-nLG3 groups. Insets show the co-localization of p-MuSK signal (in purple) with α-BTX-labelled NMJs (in red). **(B)** Analysis of the percentage of p-MuSK signal accumulation in NMJ volume after 3D reconstruction of the endplate by Imaris software, showing the increase in p-MuSK signal in ActR-Fc-nLG3 group. One-way ANOVA, F_(2,6)_=12.9; p<0.01; followed by Tuckey’s multiple comparisons test (*p<0.05; **p<0.01; ***p<0.001). N=3 animals each group, 10 NMJs each animal. **(C)** Confocal images showing ACVR-2b signal intensity in the endplates of vehicle, ActR-Fc and ActR-Fc-nLG3 groups. Insets show the co-localization of ACVR-2b signal (in purple) with α-bungarotoxin-labelled NMJs (in red). **(D)** Analysis of the percentage of ACVR-2b signal accumulation in NMJ volume after 3D reconstruction of the endplate by Imaris software. One-way ANOVA, F_(2,6)_=0.35, p>0.05 ns. N=3 animals each group, 10 NMJs each animal. Scale bars: 20 µm. Data are shown as mean ± s.e.m.

### Improved dimensions of ActR-Fc-nLG3-treated NMJs suggest an amelioration of fragile phenotype after treatment

Likewise, by aNMJ-morph we analysed the postsynaptic side (≃ 70 NMJs for each group) (Fig. 6A). ActR-Fc-nLG3 NMJs showed a reduction in fragmentation index, in particular compared to controls (one-way ANOVA; p= 0.0164) (Fig. 6B). Moreover, a significant increase in AChR clusters compared to vehicle was observed for ActR-Fc-nLG3 treated NMJs (one-way ANOVA; p= 0.0024) (Fig. 6C), indicating that more AChRs are present on the surface area. Based on these results, we then looked in more detail at a limited set of NMJs (10 NMJs per animal). Interestingly, only in ActR-Fc-nLG3-treated NMJs we observed that the surface of the synaptic cleft results amplified, to indicate sites of new outgrowth and AChR clustering (Fig. 6A, inset iii). By analysing the Feret’s diameters, we observed no differences in the maximum Feret’s diameter among the three groups (p>0.05; Fig. 6E), while the minimum Feret’s diameter resulted significantly increased in the ActR-Fc-nLG3 group compared to the other ones (one-way ANOVA; p<0.001; Fig. 6F).

Many pathways are involved in NMJ dysfunction processes, and in particular Agrin-MuSK signaling pathway has been investigated for its role in aging-related processes affecting NMJs [30]. Thus, we analysed the percentage of p-MuSK signal (volume) within the endplate (NMJ volume) (Fig. 7A, 7B) and we observed a significant increase (p<0.01) of p-MuSK signal in ActR-Fc-nLG3 compared to the other groups (Fig. 7B). In the same way, we analysed ACVR-2b signal (volume) in NMJs (volume) to look also at the myostatin inhibitor portion (ActR) of our biological (Fig. 7C, 7D). No differences were observed in ACVR-2b signal; (p>0.05; Fig. 7D), confirming that our biological mainly act on AChR clustering on the postsynaptic side by activating MuSK pathway [33], more than in regulating skeletal muscle size mediated by Activin type 2 receptor [34].

## DISCUSSION

We previously described and tested [23] a novel fusion protein, ActR-Fc-nLG3, consisting in the ActR-IIB receptor coupled to the human neuronal laminin G3 domain of agrin (nLG3) by the c-terminus of the Fc part of an Igg1 mAb [25, 35]. Based on the encouraging results we obtained on young mice [23], we investigated the effects of chronic (5 week) administration of ActR-Fc-nLG3 to aged mice, focusing on muscle mass, endurance and strength. Aging in mice reduces motor performance mimicking the loss of muscle strength and function that occurs in aged humans [30]*.* Moreover, the reduced capability of the NMJ to respond to training-induced remodelling and the age-related reduced motility have been suggested as two of the many pathogenic bases of sarcopenia [20, 36, 37]. Thus, it appeared supportive to our hypothesis to prove that correcting an impaired neuro-muscular communication would be more beneficial for motor performance, than just inducing muscle hypertrophy.

In synthesis, in the present work we mainly demonstrated that our novel compound shows its action on nerve and muscle coupling at the endplate level, supported by postsynaptic changes in NMJs by amplification of the surface area for ACh receptors. Moreover, our findings also suggest that: i) endurance can be boosted also in the aged mice, where the NMJ is defective under many aspects [30, 38]; ii) the increased endurance does not occur at the cost of losing muscle strength, which, on the contrary, is augmented at the same level of a myostatin inhibitor, despite a significantly less augmentation of muscle mass; iii) the novel biological has no effect on satellite cells, given the absence of an increased number of myonuclei after ActR-Fc-nLG3 administration.

We tested motor performances in old mice using treadmill, where equilibrium related influences are minimized [39]. The grip strength test was added to ascertain that endurance would not occur at the expenses of muscle force. The most glaring result was the significant increase of motor performance after treatment with ActR-Fc-nLG3, compared to the other treatments.

The ActR-Fc-nLG3 treated group initially had lower performance when compared to the ActR-Fc group. However, after the 5-week dosing period, the ActR-Fc-nLG3 group not only maintained the initial performance, but also showed a significant 2.82% running increase from their baseline. On the other hand, the ActR-Fc group showed a decrease in performance compared to their initial baseline.

It should also be noted that, after 5 weeks, the vehicle/PBS-treated mice had a final performance 18.27% lesser than the initial test, a likely consequence of the age-related progressive neuro-muscular deterioration that occurred in the meantime. To the best of our knowledge the only report in the literature describing significant enhancement of endurance in motor performance regarded transgenic mice overexpressing nicotinamide phosphoribosyl transferase in muscle [40]. This improvement in resistance during running was obtained only after repetitive exercise; in fact, nicotinamide alone without previous exercise did not enhance endurance. It is worth noticing that in those experiments neither average speed nor distance run were statistically different between transgenic and wild type mice. Because of these data, the authors propose elevation of nicotinamide as a means to not only increase muscle performance but also to rejuvenate most body organs [41]. Whether this is an achievable goal needs further extensive confirmation.

Moreover, the ActR-Fc-nLG3 groups started to perform significantly better soon after the beginning of the treatment, in contrast with the other treatments, suggesting a rapid effect of the novel compound on neuromuscular transmission. The proneness to become engaged in motor activity was also confirmed by the significant lower number of prodding foot shocks that the animals treated with ActR-Fc-nLG3 received. Of note, some last points to consider in the evaluation of the treadmill experiments are the intrinsic limitation imposed by the increasing speed, the time limit and the removal of the animal when not responding after 10 shocks. All these factors imposed a ceiling effect in force of which all the curves merged at the end (Fig. 2C, D) suggesting the false impression that the performance at the end of the experiment was equal for all groups.

When tested for grip strength, both treated groups (ActR-Fc-nLG3 and ActR-Fc) performed significantly stronger than the controls. Indeed, a decline in force in the PBS-treated group was observed at the end of the experiment, confirming the occurrence of progressive age-related decrement as observed also in the treadmill test. Both treatment groups show an increase in strength performance, compared to the initial levels, suggesting that this result can be mainly associated to the increase in muscle hypertrophy, known to improves muscle activity during momentary efforts [42], although not capable of sustaining prolonged activity [43, 44]. Indeed, our present results confirm that the new compound, compared to the myostatin inhibitor ActR-Fc, reduces the hypertrophying effect, but that the moderate augmentation of the body mass is due to increment of the muscle mass. In fact, the total body weight increase of about 5%, a lesser magnitude than observed for muscles mass only (10%) suggesting that the major contribution to the total body mass increase came from the muscle component. Why the addition of the neuronal agrin fragment reduces the hypertrophy caused by inhibition of the myostatin system is unknown at present. It is known that the neuronal agrin fragment alone (i.e., not the non-neuronal agrin), acting through Lrp4, activates the MuSK cascade of events essential for the organisation and structural maintenance of the NMJ [45]. Furthermore, phosphorylated MuSK is known to be capable of cross phosphorylating ErbB, leading to the activation of the ErbB pathway [46] and subsequently to the transcription and translation of NMJ proteins [47]. The role of the myostatin inhibition provided by the ActR fragment is unclear but it can help stimulate protein translation via the act/mTOR pathway [48]. Also, the agrin/LRP4/Musk pathway crosses paths with many other transducer systems [45]. Possibly in this cascade there is an interference with the muscle proteins synthesis and degradation that depends on myostatin [49].

We are planning to start disentangling these complex potential pathways exploring the activation/deactivation of reporter genes after administration of ActR-Fc-nLG3. In any case, independently of the mechanistic cause, in our view, this marginal increase obtained with the agrin-containing constructs is a significant positive achievement. In fact, as confirmed by behavioral results, whereas an excessive muscle growth may assure an increased power in a short-term performance, it may be deleterious for muscle wellbeing, especially in pathological conditions or in aging, when muscle metabolism is already deranged [43]. This is even more evident during sustained efforts and when proper innervation is lacking.

It is worth stressing that the observed enhancement of endurance was observed in old mice, whose motor performance in control conditions was declining as aging advanced.

The arrangement of myonuclei in skeletal muscle tissue has long been used as a biomarker for muscle health [50]. ActR-Fc-nLG3 has no major effect on muscle fibers, as confirmed by the significant lower number of peripheral nuclei per fiber than the other groups. The increase in nuclei in ActR-Fc group, on the contrary, seems to be reciprocally related to the muscle fiber enlargement (evaluated by CSA analysis), as also seen in previous papers [51;52]; indeed, hypertrophy can lead to a lasting elevated number of myonuclei, the nuclei in turn can sustain the hypertrophic phenotype [51], providing resistance only in short-term efforts. In support of this hypothesis, we also observed a modification in the muscle type proportions following the different treatments. Indeed, in ActR-Fc treated muscles we observed a significant switch from oxidative to glycolytic phenotype compared to the other groups, that is in line with the limited ability to endure prolonged exertion. ActR-Fc-nLG3 treated muscles show an intermediate phenotype between control and ActR-Fc fibers: the treatment has a modest effect on glycolytic fibers and lead to a higher percentage of oxidative fibers than ActR-Fc treatment.

The observations that the anti-myostatin induced hypertrophy was quenched by our biological, and that no major histological changes were observed in ActR-Fc-nLG3 muscle fibers, led us to postulate that the new compound could act at NMJ level. ActR-Fc-nLG3 preserves innervation at NMJ level, confirming the efficacy of this novel biological in reinforcing nerve-muscle interaction. Indeed, for ActR-Fc-nLG3 animals we observed a higher percentage of innervated endplates, showing a stronger NF signal intensity, an increased number of presynaptic branch points, and the consequent decreasing in denervated and fragmented NMJs. Alteration in morphologic remodelling in aged NMJs results in more fragmented, damaged or denervated endplates, with greater spatial uncoupling between ACh vesicles and ACh receptor clusters [53], that may contribute to neuromuscular dysfunction in aging. In control conditions, the typical fragmented appearance of elder NMJs was evident [54]. The strength in nerve-muscle connections in ActR-Fc-nLG3 group and the reduction of fragmentation are in line with the amplification of surface area for ACh receptors observed in the NMJs of these mice. Indeed, we confirmed the capability of ActR-Fc-nLG3 compound to induce postsynaptic changes on the NMJ, as previously observed in younger mice [23]. NMJs size were significantly increased (nearly doubled) after ActR-Fc-nLG3 treatment compared to vehicle condition. ActR-Fc-nLG3-treated NMJs are also bigger than those of ActR-Fc group, with a higher complexity index and a reduced fragmentation index, confirming the role of nLG3 fragment in maintaining the neuronal fiber-endplate coupling. The postsynaptic changes induced by ActR-Fc-nLG3 also include enfolded junctional folds of the motor endplate by amplification of the surface area for ACh receptors (already observed in young ActR-Fc-nLG3 treated mice [23]) as a sign of an increase in the contact surface between the nerve terminal and the endplate. Moreover, the morphological analysis on NMJs suggests that the loss of AChR clusters in aging might occur specifically from one side of the NMJ or from two opposite sides of the endplate (e.g., ‘north’ and ‘south’, ‘east’ and ‘west’ sides, rather than from a combination of ‘north’ and ‘west’), which would account for the observed change in Feret min while leaving Feret max unaffected. This observation implies that NMJ loss is not a completely random occurrence but rather may exhibit some degree of organization.

These findings could also provide an explanation for the remarkable increase in endurance during treadmill exercise in ActR-Fc-nLG3 treated mice. Moreover, the main effect of ActR-Fc-nLG3 on NMJs and nerve coupling is also supported by the significant increase in phosphorylated MuSK tyrosine kinase signal in the endplates, that suggests, as reported above, the activation of the pathway known to play a role in AChR clustering in the postsynaptic side and in inducing nerve terminal differentiation in the presynaptic portion [33; 55]. On the contrary, no differences between groups have been observed in the signalling through activin receptors (ACVR-2b) that regulates skeletal muscle mass [56-58], further suggesting that our novel biological preferentially acts on nerve and endplate crosstalk. These data represent preliminary observation on molecular pathways required after ActR-Fc-nLG3 administration; in future studies we intend to delve into further molecular analyses to unravel the specific mechanisms involved.

Despite the other parameters remaining unaltered, a slight increase (44.2%) in ActR-Fc NMJ area was also observed compared to vehicle. This increase could be coupled to muscle fiber growth and the consequent expansion of the postsynaptic muscle fiber membrane and ACh receptors spreading apart, as reported previously in other muscles [59].

Further investigation employing electrophysiological read-outs will be warranted to validate the compound's efficacy in preventing or slowing down denervation, thereby preserving neuromuscular functionality with irrefutable evidence.

In conclusion, to the best of our knowledge this study is the first to highlight the capability of a novel biological to act at the neuromuscular level for the restoration of the crosstalk between neurons and muscle, the amelioration of atrophic muscle phenotype and the improvement in motor performance. Moreover, our compound is particularly efficient in the elderly, where motor function undergoes age-related progressive impairment [30, 60]. We deem those results to be an indirect support of our hypothesis that muscle hypertrophy per se is not sufficient to improve sustained muscle performance without additionally addressing the innervation component. Altogether, these data raise hope for a novel approach to the palliative treatment of sarcopenia and other neuromuscular disorders (e.g., Amyotrophic Lateral Sclerosis, Spinal Muscular Atrophy, the Muscular Dystrophies among others) that share a combined myo- and neurogenic component.

## Data Availability

The datasets used and/or analysed during the current study are available from the corresponding author, Roberta Schellino, upon reasonable request.
